# Total Proximal Interphalangeal Joint Arthroplasty as a Treatment for Advanced Boutonniere Deformity of the Finger

**DOI:** 10.7759/cureus.106317

**Published:** 2026-04-02

**Authors:** Jakub Florek, Patryk Kawa, Filip Georgiew, Oles Petrovych, Krystian Mleczko

**Affiliations:** 1 Department of Orthopaedics and Traumatology, Rydygier Hospital, Brzesko, POL; 2 Faculty of Health Science, University of Applied Science, Tarnów, POL; 3 Medical and Rehabilitation Centre, Reha Medica, Tarnów, POL

**Keywords:** boutonniere deformity, degenerative disease, hand, pip, proximal interphalangeal joint, surgical treatment, total arthroplasty

## Abstract

Boutonniere deformity results from damage to the central band and structures stabilizing the finger's extensor apparatus, leading to flexion of the proximal interphalangeal (PIP) joint and hyperextension of the distal interphalangeal (DIP) joint. Untreated or delayed diagnosis of boutonniere deformity results in progression of both deformity and degenerative changes in the PIP joint. In advanced, chronic deformities (Burton IV), surgical treatment includes arthrodesis or PIP joint replacement. In the presented case, a 29-year-old right-handed man presented with a persistent boutonniere deformity of the fifth finger of his right hand and pain that limited his function. The patient had two finger injuries, 13 and 11 years earlier. X-rays of the injuries revealed no fractures, and the finger was immobilized. After the immobilization of the second injury was removed, the patient experienced mild pain and observed a gradual worsening of the finger deformity. Upon admission to the orthopedic clinic, examination revealed 55° of flexion in the PIP joint, 30° of hyperextension of the DIP joint, complete lack of motion in the PIP joint. Mobility in the DIP joint was complete. X-rays revealed advanced degenerative changes in the PIP joint. The patient underwent implantation of a cementless, semi-constrained PIP endoprosthesis (Interphalangeal Proximal Prosthesis (IPP2); 3S Ortho, Lyon, France) and central band reconstruction. Postoperative immobilization lasted six weeks, followed by intensive rehabilitation. Fifteen weeks after surgery, 50° of flexion and a 5° of extension deficit were achieved in the PIP joint, with full DIP mobility. After 21 weeks, the PIP joint flexion range increased to 85°, and the pain completely resolved. The patient returned to full manual dexterity and physical activity. Chronic boutonniere deformity can lead to progression of PIP joint arthrosis and significant impairment of hand function. PIP joint arthroplasty combined with extensor reconstruction is a valuable alternative to arthrodesis in patients requiring preserved mobility and grip precision. A properly selected treatment method and early, intensive rehabilitation allow for excellent functional outcomes.

## Introduction

Boutonniere deformity involves flexion of the finger at the proximal interphalangeal (PIP) joint and simultaneous extension at the distal interphalangeal (DIP) joint. The deformity usually results from a finger injury that disrupts the continuity of the central band and triangular ligament, resulting in palmar displacement of the lateral bands of the extensor tendon. The most common mechanism of injury is an axial impact to the tip of the extended finger. Boutonniere deformity also frequently occurs in patients with rheumatoid arthritis [1].

The most commonly used classification of boutonniere deformity is the Burton classification, which is based on the PIP joint compliance and the presence of degenerative changes in the joint [2]. Diagnosis of acute central band injury is possible based on a thorough history, consideration of the mechanism of injury, and a dedicated clinical test (Elson's test) [1]. Currently, there are no guidelines for the standard treatment of boutonniere deformity [3,4]. Treatment of acute central band injury usually involves immobilization of the PIP joint in extension.

Surgical treatment is used when the central band is completely damaged, a significantly displaced bone fragment of the middle phalanx is present, or conservative treatment fails to improve. Surgical treatment involves reinsertion of the finger's extensor apparatus. Inappropriately treated or undiagnosed central band injuries lead to progressive deformity and subsequent degenerative changes in the finger joints. In cases of persistent deformities, surgical treatment should be considered, including arthrodesis or PIP joint replacement [1]. In this case report, we present a case of persistent boutonniere deformity of the right fifth finger with advanced degenerative changes of the PIP joint (Burton grade IV) treated with cementless, semi-constrained, total PIP joint arthroplasty and central band replacement of the right fifth finger.

## Case presentation

This paper presents the case of a 29-year-old right-handed manual laborer who presented to the Trauma and Orthopedic Surgery Department due to a deformity and painful limitation of mobility in the fifth finger of his right hand. The patient had sustained two injuries to his finger. The first injury occurred at the age of 16 when he was hit by a ball while playing soccer. He then went to the emergency department, where an X-ray revealed no fracture, and the finger was immobilized in a splint for several weeks. After the immobilization was removed, the patient experienced no pain and no deformity. The second injury was also caused by a ball. After reporting to the emergency department, another X-ray revealed no fractures, and the finger was immobilized, but this time only for a few days. After the immobilization was removed, the finger deformity slowly worsened, and the patient experienced moderate pain during intensive use of the finger. Despite the functional limitations and cosmetic defect, the patient did not consult a physician.

**Figure 1:**
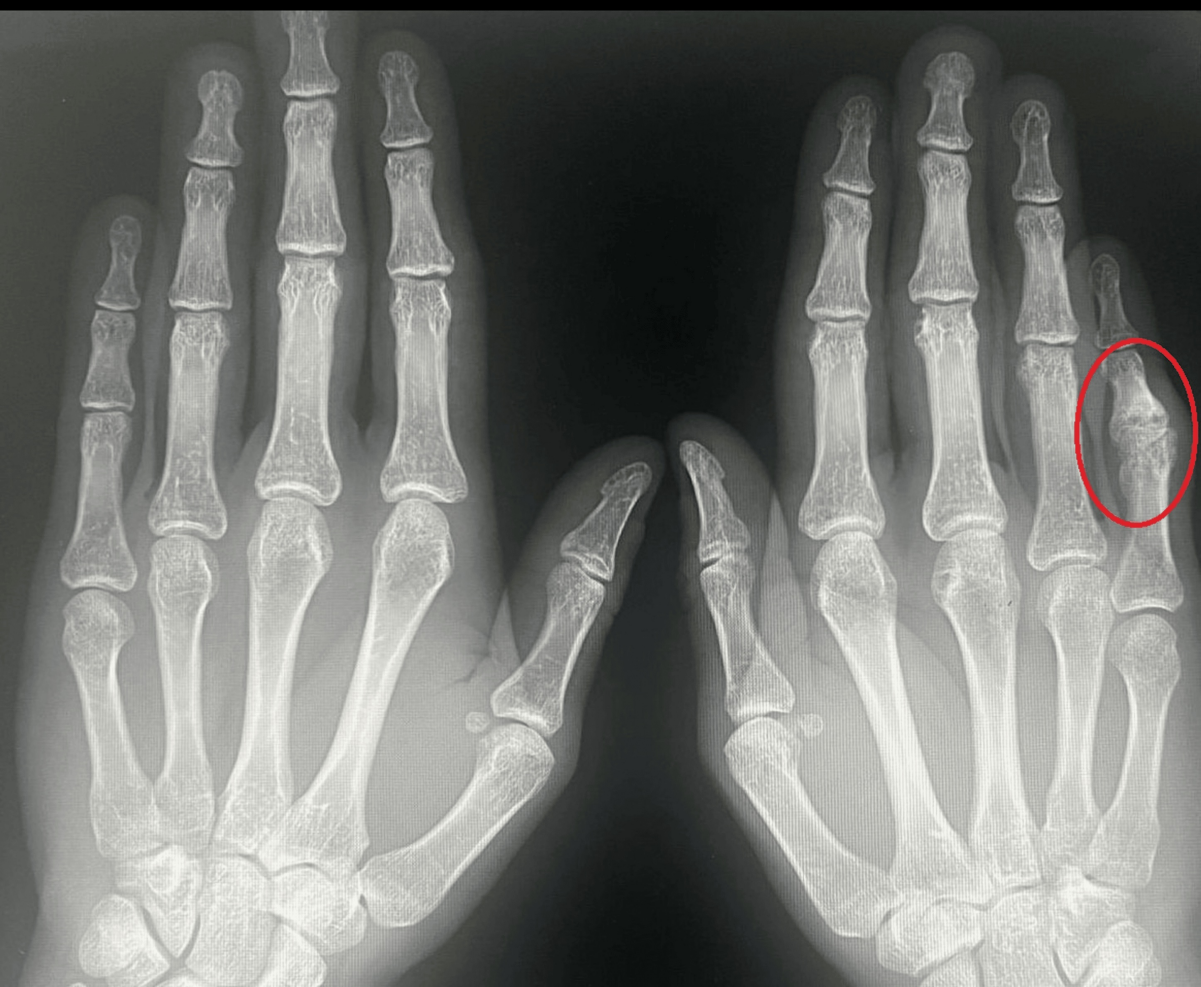
X-ray of both hands before surgery in AP projection Red circle indicates the proximal interphalangeal joint. AP: anteroposterior.

**Figure 2:**
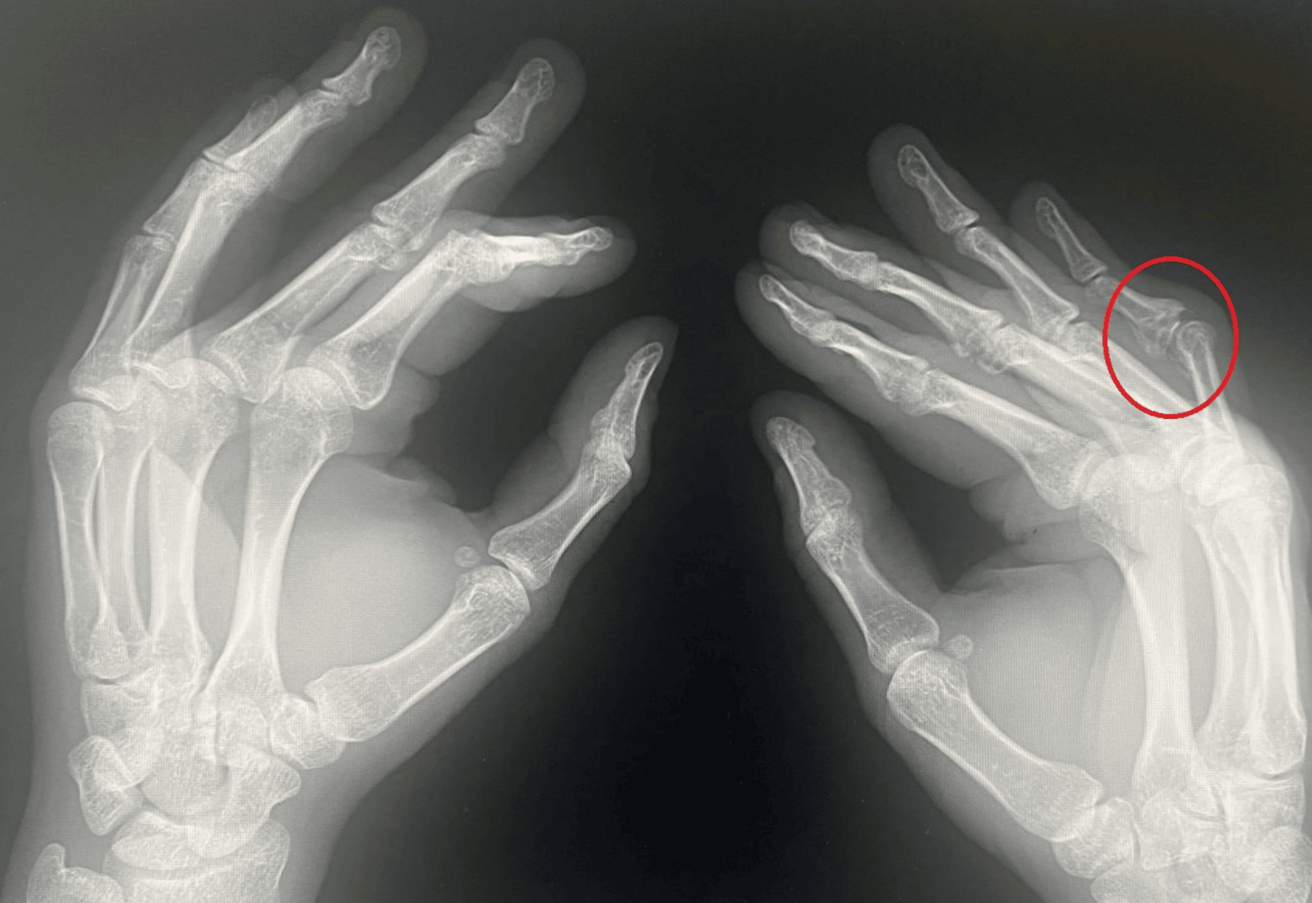
X-ray of both hands before surgery in lateral projection Red circle indicates the proximal interphalangeal joint.

At the age of 29, due to painful limitation of finger mobility (3/10 on the Numerical Rating Scale (NRS)) and pressure from his partner, the patient decided to seek specialist help [5]. Physical examination revealed a persistent boutonniere deformity of the fifth finger of the right hand (55° of flexion in the PIP joint and 30° of hyperextension in the DIP joint). Mobility in the PIP joint was completely lost, and the joint was locked in a flexion contracture position (fixed deformity), while mobility in the DIP joint was complete. X-ray examination revealed advanced degenerative changes in the PIP joint of the fifth finger of the right hand (Figures 1, 2). Blood supply, thermal insulation, and innervation of the finger were normal.

**Figure 3:**
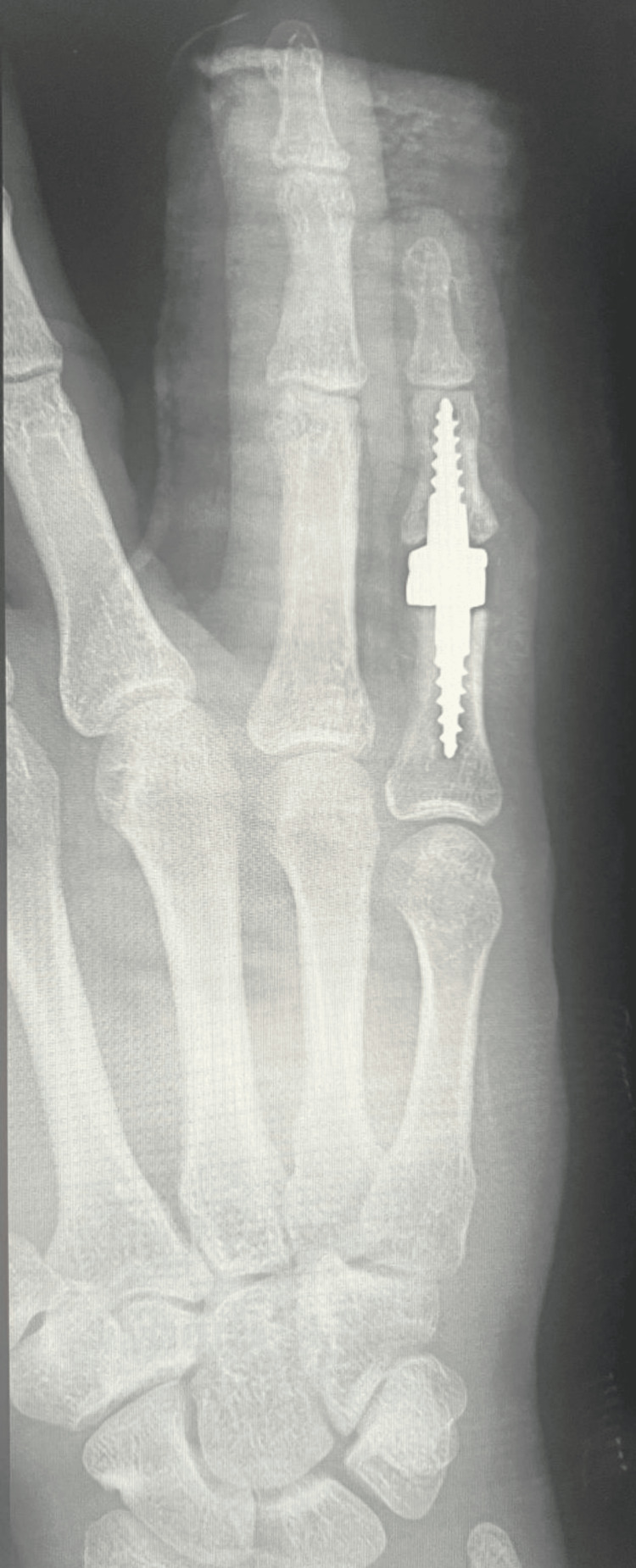
X-ray of the right hand in the AP projection on the first day after surgery AP: anteroposterior.

**Figure 4:**
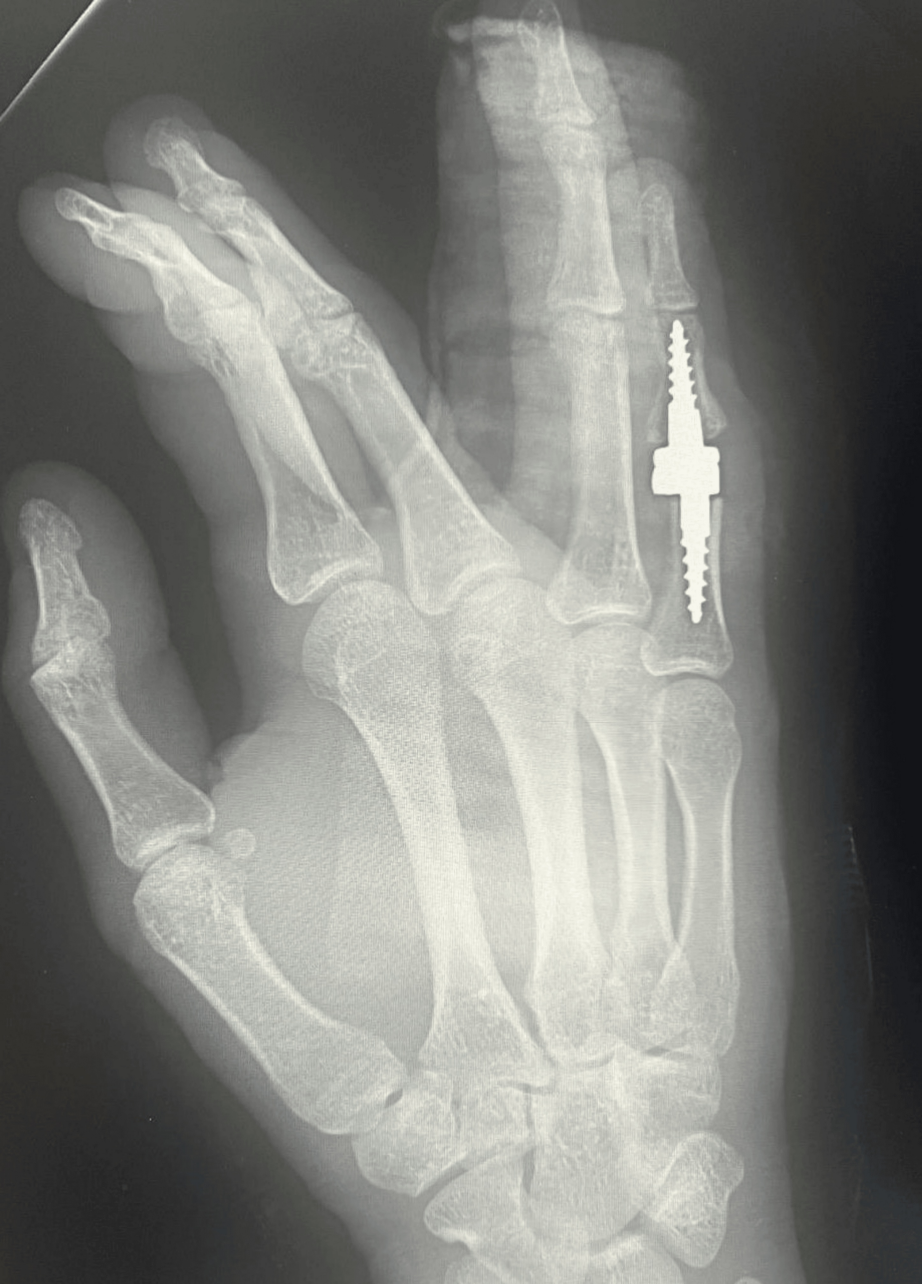
X-ray of the right hand in the lateral projection on the first day after surgery

Due to advanced finger deformity and accompanying degenerative changes (Burton IV), the patient underwent implantation of a cementless, semi-constrained Interphalangeal Proximal Prosthesis (IPP2) endoprosthesis (3S Ortho, Lyon, France), and central band repair of the right fifth finger (Figures 3, 4). After surgery, the right fifth finger was immobilized in full extension in a plaster splint.

**Figure 5:**
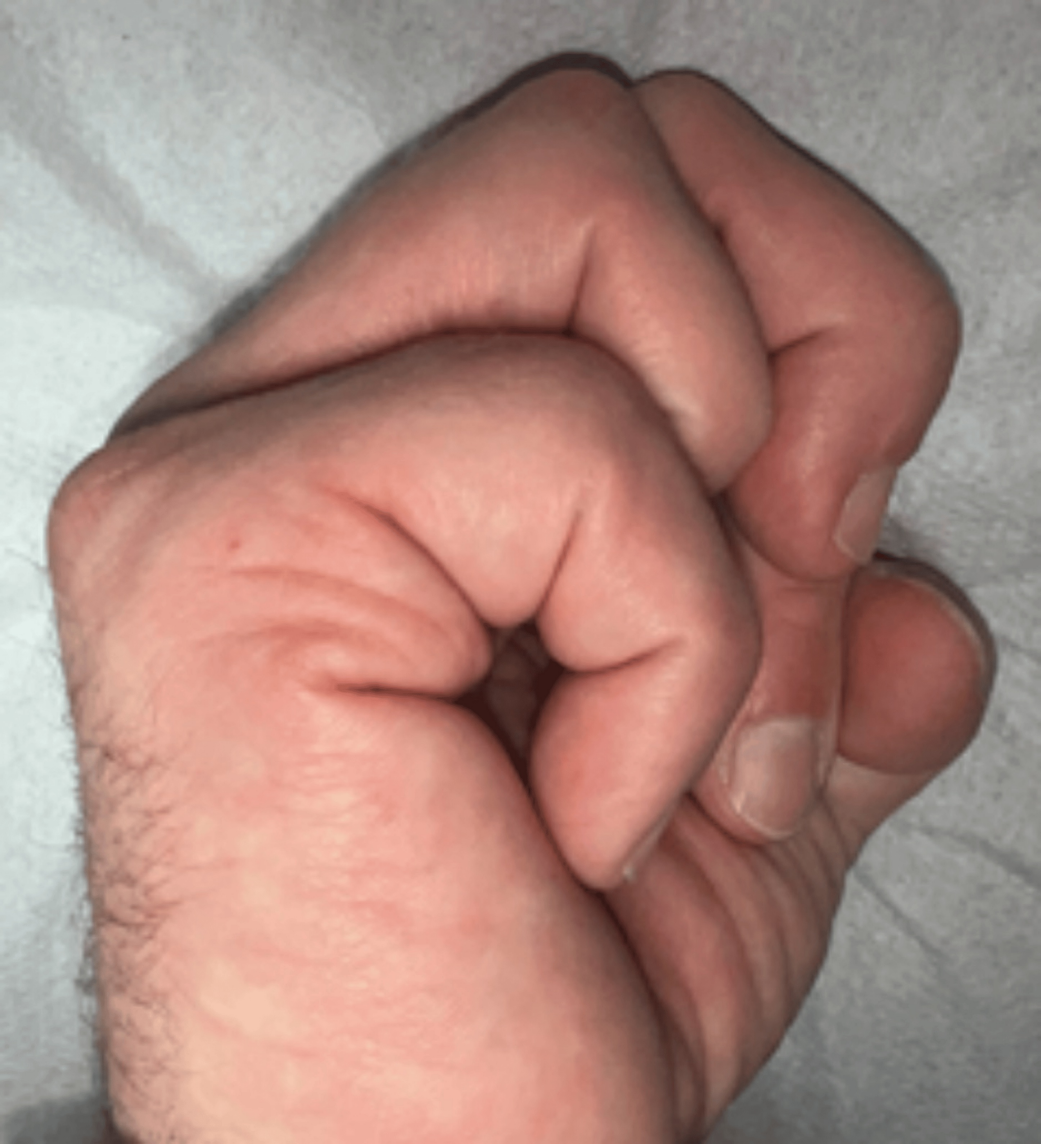
Position of the fifth finger of the right hand in a position of possible full flexion at week 21 of the follow-up

**Figure 6:**
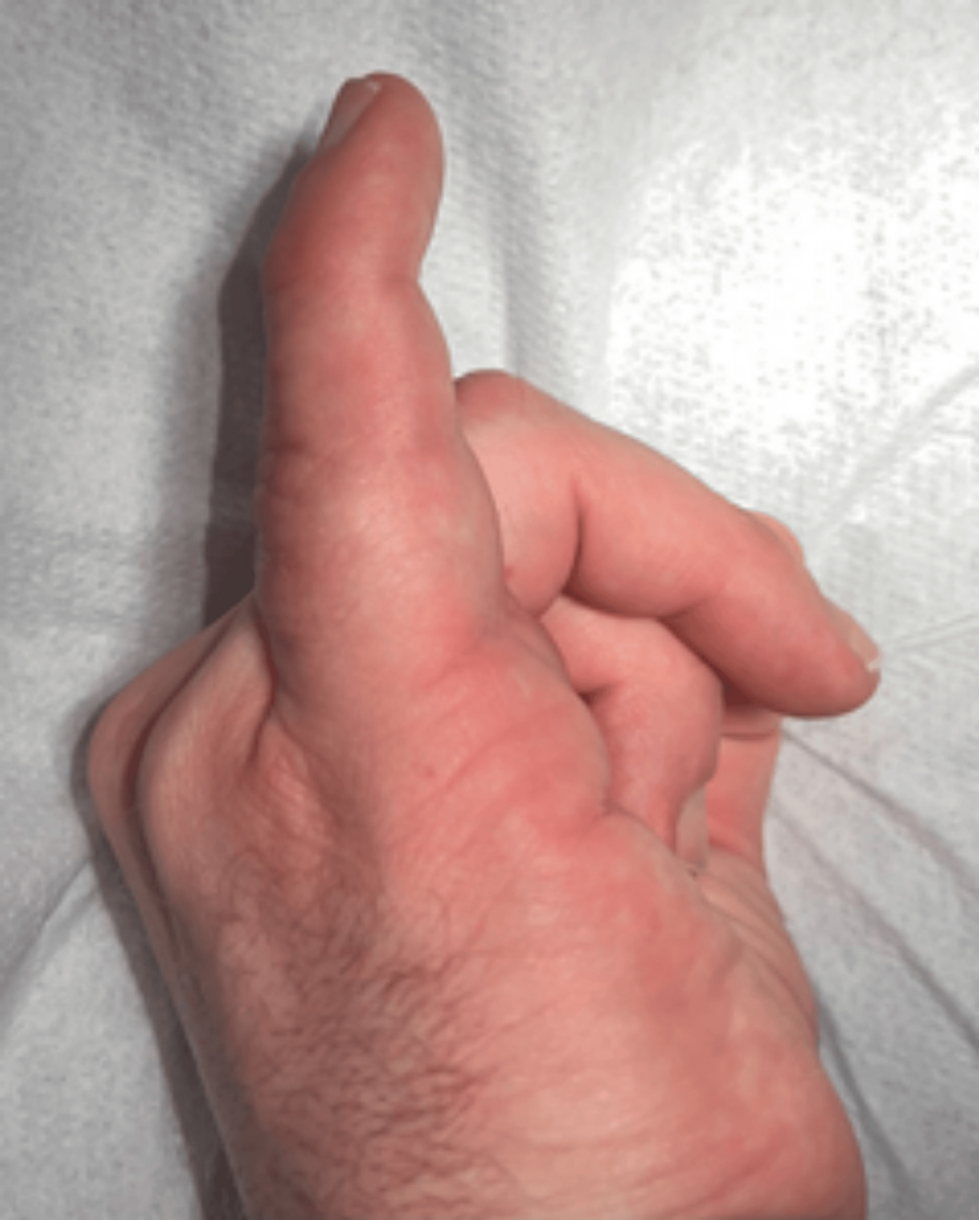
Position of the fifth finger of the right hand in a position of possible full extension at week 21 of the follow-up

In the first week after surgery, the patient reported pain at a level of 1/10 on the NRS scale. He had no fever and complied with postoperative recommendations. After three weeks, the patient continued to complain of pain at the same level (1/10 on the NRS scale), and the postoperative wound healed normally (sutures were removed after 10 days). Rehabilitation began, which included active exercises of the entire operated limb, avoiding the joints subjected to immobilization. Additionally, a low-frequency pulsed magnetic field was introduced to accelerate healing. A total of 20 treatments were performed, each lasting 20 minutes, five times per week, with rectangular pulses. During a six-week follow-up at the Orthopedic Clinic, the cast was removed and a follow-up X-ray was performed, revealing a properly implanted PIP joint endoprosthesis in the fifth finger of the right hand. The postoperative scar was pale. Finger mobility was limited, 20° of flexion with full extension in the PIP joint and full extension and flexion in the DIP joint. At this stage of treatment, the patient was referred for specialized rehabilitation, which included whirlpool massage of the limb (before exercises), post-isometric muscle relaxation, and stretching exercises. Additionally, the patient received a home exercise program that included flexor tendon gliding exercises, self-performed stretching exercises, and limb activation during daily activities. Fifteen weeks after surgery, the patient achieved 50° of flexion with a 5° extension deficit in the PIP joint and full range of motion in the DIP joint. Pain intensity at both rest and activity was 0/10 on the NRS scale. Continued rehabilitation resulted in further clinical improvement. At week 21, PIP flexion improved to 85°, representing approximately 75%-85% of normal range. This degree of motion is sufficient for most activities of daily living and manual tasks (Figure 5). During this period, a decrease in the extension range of motion of the DIP joint was observed. The DIP joint showed a 5° passive extension range of motion deficit and a 15° active extension deficit (Figure 6). The flexion range of motion in the joint, similar to the previous observation periods, was 70°.

To describe quantitative abnormalities in the range of motion in a joint, the following concepts can be used: stiff, which means a limited range of motion, but the joint still has some mobility (movement is limited, but exists); ankylosed, i.e., pathological stiffening (complete lack of movement); and fixed deformity, a condition in which the joint is locked in a specific, incorrect position (flexion/extension, complete lack of movement). Detailed values ​​of the observed parameters in the individual observation periods are presented in Table 1.

**Table 1:** Results of the assessed parameters in subsequent observation periods NRS: numerical rating scale; PIP: proximal interphalangeal joint; DIP: distal interphalangeal joint. Stiff, which means a limited range of motion, but the joint still has some mobility (movement is limited, but exists). Fixed deformity, a condition in which the joint is locked in a specific, incorrect position (flexion/extension, complete lack of movement).

Treatment stage	NRS [5]	PIP range of motion (extension/flexion)	DIP range of motion (extension/flexion)
Before	3/10	Fixed deformity of the joint in the flexion position of 55°	Hyperextension 30°, full range of flexion 70°
6 week	0/10	Flexion range 20° (stiff), full extension 0°	Full range of flexion 70°, full extension 0°
15 week	0/10	Flexion range 50° (stiff), extension deficit 5° (stiff)	Full range of flexion 70°, full extension 0°
21 week	0/10	Flexion range 85°, full extension 0°	Full range of flexion 70°, extension deficit 5° (stiff)

**Figure 7:**
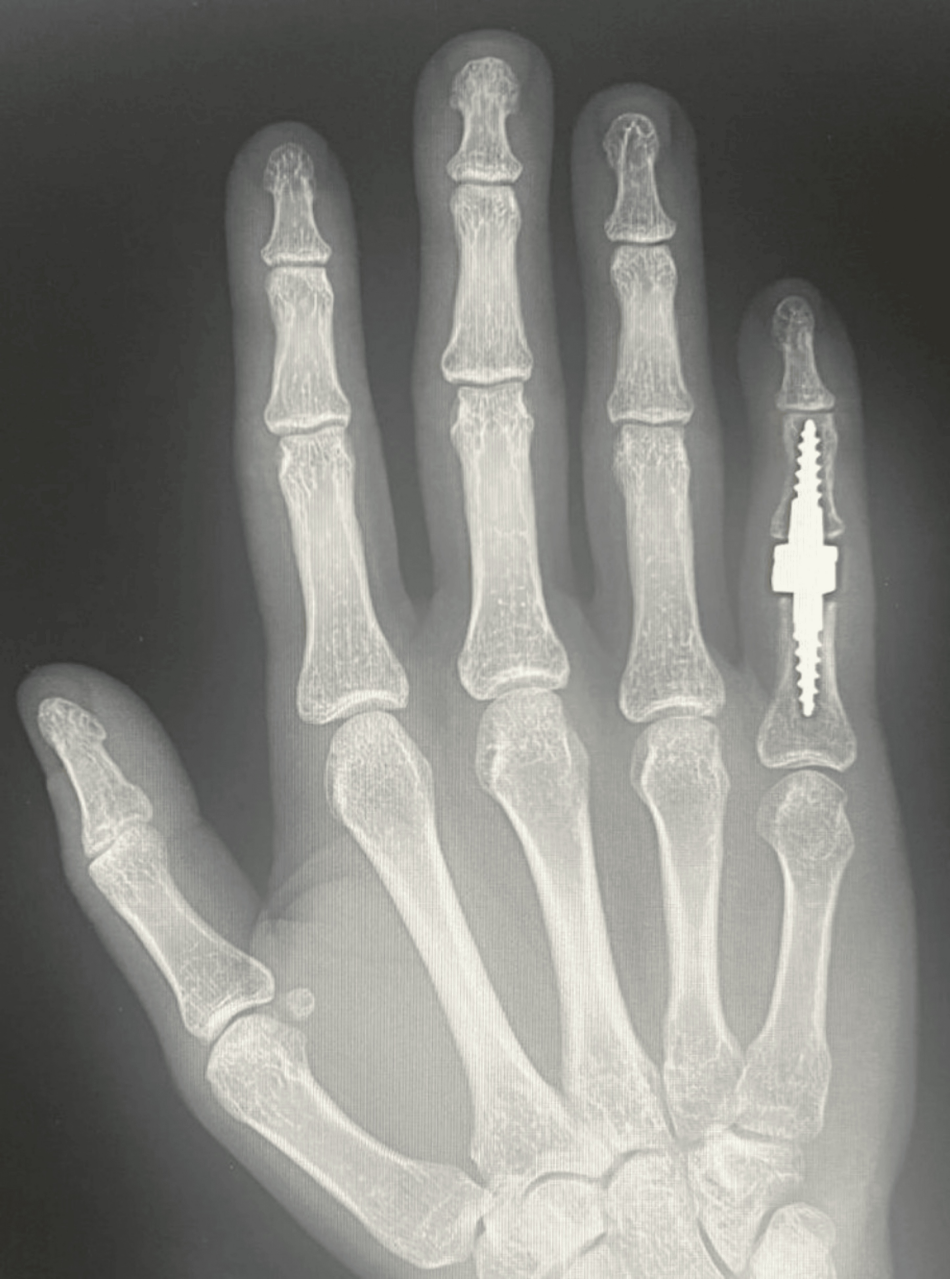
Right hand X-ray in AP projection at week 21 of follow-up

**Figure 8:**
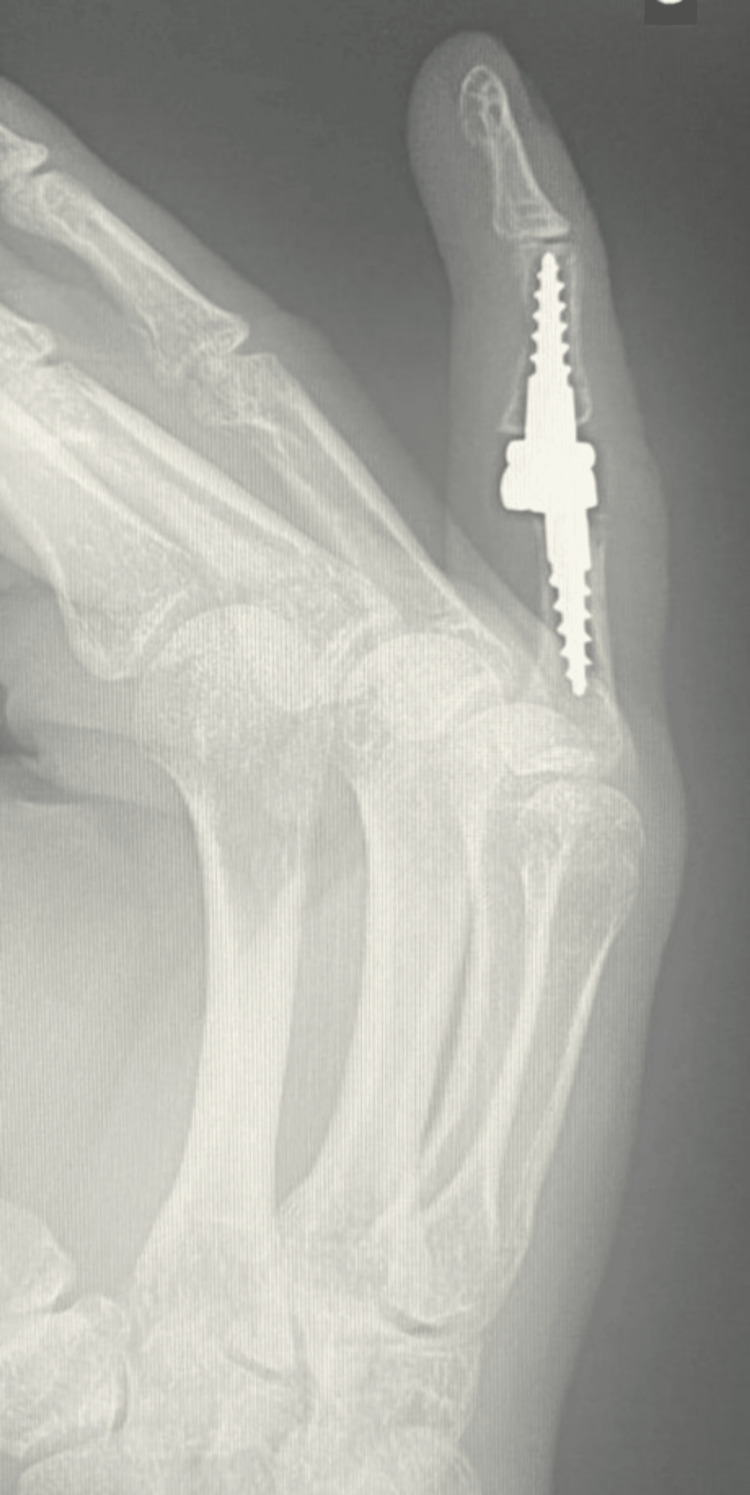
Right hand X-ray in lateral projection at week 21 of follow-up

The last follow-up radiograph taken at week 21 of follow-up showed the correct positioning of the prosthetic components (Figures 7, 8).

## Discussion

Central band injuries are relatively rare, which is why there are few publications on the subject in the medical literature. These are usually single clinical cases illustrating an isolated case and treatment method [6]. Due to the lack of larger studies, there are no standardized treatment protocols for central band injuries and posttraumatic boutonniere deformity. Therefore, hand surgeons must often rely on their own experience, that of their senior colleagues, and isolated reports in the literature [3,4].

Boutonniere deformity usually does not appear immediately after the injury, but only after several to a dozen weeks. Patients often only then seek medical attention. Due to the low prevalence of central band injuries and the late average time of the first medical visit, it is important to be aware of the possibility of this type of injury so that appropriate therapeutic measures can be taken early to reduce the risk of deformity progression. In his study, Souter presented the cases of 11 patients aged 11 to 58 years with posttraumatic boutonniere deformity who abandoned treatment (conservative and surgical) [7]. Two of these patients experienced spontaneous improvement, three experienced persistent deformity, two experienced slight worsening, and three experienced significant progression of the changes [7].

As mentioned above, there are no standardized protocols for the conservative treatment of central band injuries/boutonniere deformity. There are many different finger immobilization methods, the common and key element of which is immobilization in extension of the PIP joint. The decision to immobilize the DIP and metacarpophalangeal (MP) joints should be made after considering the degree of deformity/injury, coexisting injuries, patient compliance, and the physician's individual experience. Regardless of the type of orthosis used, it should be emphasized that a period of immobilization of at least four to six weeks is necessary to achieve a good result [7]. In our case, the period of finger immobilization following the injuries was likely crucial. After the first injury, the finger was immobilized for several weeks; after removal of the immobilization, the patient experienced no symptoms. After the second injury, the immobilization was removed after several days. This immobilization period may have been too short for proper healing of the central band injury.

The literature provides a variety of surgical treatment options for acute injuries, ranging from immobilization of the PIP joint with Kirschner pins in patients with closed injuries unsuitable for 24-hour immobilization, to primary reinsertion of the central band to the base of the middle phalanx using an anchor in patients with open injuries. Avulsion fracture of the middle phalanx with avulsion of the central band is also possible [7]. Patients with persistent boutonniere deformities typically retain full flexion and grip strength and have few functional limitations of the hand. Therefore, the decision to surgically treat this type of deformity should always be made by the patient with the awareness of possible deterioration of hand function, including extension contractures of the PIP joint. There are many surgical treatment options for persistent boutonniere deformities. Before surgery, the patient should achieve full extension of the PIP joint using a brace. The goal of surgical treatment is to achieve tendon rebalancing. This can be achieved through methods such as distal Fowler or Dolphin tenotomy, which removes excessive extension forces in the DIP joint, mobilization and suturing of the lateral bands in the midline, distal to the PIP joint (Littler and Eaton technique), or multi-stage reconstruction [8].

Neglected, untreated damage to the central band and the PIP joint area may lead to the worsening of the boutonniere deformity and progression of degenerative changes in the PIP joint, which may cause severe pain in the late stage [7,9]. Contractures of the palmar plate and collateral ligaments, intra-articular fibrosis, and concomitant degenerative changes in the proximal interphalangeal joint were classified by Burton as grade IV boutonniere deformity [2]. For this type of deformity, the treatment of choice is arthrodesis of the PIP joint in the functional position due to persistent soft tissue contractures [10]. Arthrodesis of the PIP joint reduces pain and eliminates the deformity, but limits precise finger movements and, secondarily, manual dexterity. In our patient's case, the goal was to restore full hand function with the ability to perform fine movements. Therefore, aware of the risks, we opted for PIP joint arthroplasty.

In the presented case, in addition to informed qualification and subjecting the patient to a complex surgical procedure, the appropriate timing of postoperative management was also crucial. After the procedure, the finger remained immobilized in a plaster splint for six weeks to ensure optimal healing conditions for the repaired central band and osseointegration of the endoprosthesis. Such prolonged immobilization could have resulted in postoperative stiffness of the finger, but too rapid a return to movement could have resulted in damage to the plastic surgery of the finger's extensor apparatus. After the immobilization was removed, the patient underwent an intensive finger rehabilitation protocol, which allowed for a gradual return to virtually full range of flexion/extension in the PIP and DIP joints, without axial disturbances, deformities, or pain. Thanks to this treatment outcome, the patient was able to return to full manual dexterity and physical activity, significantly improving his quality of life.

Following endoprosthesis implantation, the risk of complications should always be considered. These can be divided into early (postoperative) and long-term complications, which include implant failure, infection, aseptic loosening, periprosthetic fractures, dislocations, and joint contractures. Darwish et al. reviewed the literature published between 1990 and 2021 on PIP joint arthroplasty [11]. The reported complication rates were dependent on the type of prosthesis (material used) and were 11.3% for silicone implants, 18.5% for pyrocarbon prostheses, and 22.4% for metal prostheses [11]. A meta-analysis conducted by Forster et al. showed no statistically significant differences between the complication rates and implant types [12]. The complication rates were 11% for silicone, 14% for pyrocarbon, and 10% for metal-polyethylene prostheses [12].

It is also worth mentioning that each endoprosthesis has a defined survival time, which carries the risk of requiring revision surgery in the future. Wagner et al. report that PIP joint arthroplasty procedures achieve approximately 75% survival after 14 years, but 25% require revision surgery [13]. According to the authors, the need for revision surgery may affect up to 30% of patients after a five-year follow-up [13]. The design and material of the prosthesis certainly influence implant survival. Herren and Reischenböck et al. report that resurfacing prostheses made of various materials, such as pyrocarbon, titanium, or ceramic, have a higher rate of postoperative complications [10,14]. Silicone implants are characterized by poorer postoperative stability and a higher failure rate [10,14].

A commonly used technique for treating posttraumatic deformities is arthrodesis. Its goal is pain reduction combined with sufficient global hand function. In cases of significant joint deformity and/or existing instability, arthrodesis tends to be recommended, as an unstable prosthesis is prone to failure. In such cases, joint fixation provides reliable results. The three most frequently used techniques are K-wires, tension bands, and compression screws. K-wires still have their place in acute trauma with soft tissue defects or replantation [15]. Compared to arthrodesis, implanting a PIP joint replacement certainly requires a greater financial outlay and is not always successful. Arthroplasty provides a greater range of motion compared to arthrodesis, but patients undergoing arthroplasty may lose some mobility after surgery. Furthermore, the choice of this treatment option depends on the surgeon's preferences, experience, and capabilities. Vitale et al. suggest that the decision for prosthetic arthroplasty versus arthrodesis in the index finger of patients with osteoarthritis or posttraumatic arthritis should be made with patient goals in mind and in light of greater risk of complications associated with arthroplasty [16]. There was a 4.3 times increased risk of complication in patients undergoing arthroplasty versus arthrodesis. There were no differences in pain relief, satisfaction, or Michigan Hand Questionnaire scores between treatment groups. The biggest advantage of arthroplasty is the ability to restore full range of motion [16]. Wagner et al. report that risk of revision surgery was associated with younger age [17]. The 10-year implant survival rate was 72% for the patients younger than 60 years versus 86% for those older than 60 years. The most common complication in young patients was dislocation [17]. According to Luther et al. in symptomatic post-traumatic or idiopathic PIP arthritis, PIP joint replacement is a recognized alternative to joint arthrodesis, leading to good range of motion and pain relief in selected cases [18]. Due to the noticeable complications and reoperation rate, one could consider PIP arthroplasty in young, physically demanding patients to be a relative contraindication [18]. According to Harris et al., patients are more likely to prefer the arthroplasty method due to ability to preserve joint motion and grip strength, relative to those associated with arthrodesis, which are characterized by lower costs, decreased need for reoperation, and shorter reoperation times [19].

## Conclusions

This case demonstrates that inadequately treated central band injury can lead to advanced boutonniere deformity with progression of PIP joint arthrosis. Total PIP joint arthroplasty with simultaneous central band repair can be a method for reducing deformity and restoring normal finger function. In appropriately selected patients, a cementless, semi-constrained endoprosthesis provides excellent pain reduction, stable reconstruction, and restoration of joint mobility. Early rehabilitation is crucial for optimal recovery of the operated finger and hand function.
